# A synthetic dataset for time series super-resolution with deep learning

**DOI:** 10.1038/s41597-026-07344-7

**Published:** 2026-05-02

**Authors:** Julio Ibarra-Fiallo, D’hamar Agudelo-Moreno, Juan A. Lara

**Affiliations:** 1 Colegio de Ciencias e Ingenierías, Universidad San Francisco de Quito, Cumbayá, Ecuador; 2 Universidad de Córdoba, Córdoba, Spain

**Keywords:** Databases, Education

## Abstract

The increasing application of time-series analysis in fields like biomedical engineering or telecommunications emphasizes the need for high-quality data to train and evaluate advanced machine learning models. Acquiring temporal data at suitable resolutions is often limited by ethical, economic, or practical constraints. We introduce CoSiBD (Complex Signal Benchmark Dataset for Super-Resolution), a synthetic dataset designed for reproducible time-series super-resolution research. CoSiBD provides 2,500 high-resolution signals (*N* = 5, 000 samples each over a reference domain *τ* ∈ [0, 4*π*]) with aligned low-resolution versions at four levels (150, 250, 500, and 1,000 samples) obtained via uniform decimation. Signals are generated with diverse non-stationary behaviors through piecewise frequency modulation and spline-based amplitude envelopes, and provides both clean and noisy variants. Signals are distributed as NumPy arrays, plain text, and JSON, with comprehensive metadata describing segment structure, generation parameters, and seeds for full reproducibility. Technical validation analyzes spectral properties and reports baseline SR benchmarking and transfer experiments on EEG and speech data.

## Background & Summary

The analysis and simulation of temporal signals are fundamental across science and engineering. These techniques provide critical insights into dynamic processes in multiple domains. For instance, in biomedical research, physiological time series such as electroencephalography (EEG) and electrocardiography (ECG) support the study of neural and cardiac function^1–3^. Another example is telecommunications, which rely on signal processing to ensure data fidelity across noisy media^4^. Developing robust tools for interpreting time-varying data continues to support both scientific discovery and practical applications.

Recent advances in deep learning have contributed significantly to this field by enabling automatic extraction of complex features from raw signals. Deep learning approaches, such as Convolutional Neural Networks (CNNs) or Generative Adversarial Networks (GANs) have demonstrated improved performance over traditional techniques in image, speech, and time-series processing tasks^5,6^. These models support fine-grained signal reconstruction, allowing researchers to explore temporal dynamics in new ways.

Despite this progress, deep learning methods for temporal signal processing often require large quantities of labeled, high-quality data. Access to such data is frequently constrained. For instance, in medicine, data access is constrained by medical privacy regulations such as the General Data Protection Regulation (GDPR) and the Health Insurance Portability and Accountability Act (HIPAA), necessitating the development of synthetic data approaches to support clinical research^7^. In other domains, including telecommunications, data availability is limited by proprietary protocols and the high cost of acquiring large-scale channel sounding datasets for diverse environments^8^. These limitations are particularly relevant in super-resolution (SR) tasks, where models require paired low- and high-resolution signals for effective training. Here, temporal SR refers to reconstructing a higher-sample-count (higher temporal resolution) discrete sequence from a uniformly decimated low-resolution version.

This task has broad potential. In biomedical monitoring and sensing, for instance, SR can help reconstruct higher-resolution physiological time series (e.g., EEG), potentially improving the analysis of subtle physiological irregularities^3^. SR also applies to audio/speech enhancement and telecommunications (e.g., neural audio upsampling and bandwidth extension, relevant to telephony and compression)^9,10^.

Deep learning offers adaptive alternatives to these traditional methods. However, the lack of accessible, high-quality paired datasets remains a significant barrier to progress in this area.

To support research in super-resolution for time-series data, we present the Complex Signal Benchmark Dataset (CoSiBD). CoSiBD is a synthetic dataset composed of time-series signals with variable resolution, frequency characteristics, and noise levels. Our dataset is intended with a double purpose: a) to provide a resource for training and evaluating deep learning SR models under controlled, reproducible conditions, which can constitute a sort of benchmark for this problem; and b) a resource for training deep learning models to be used for SR. It includes non-stationary, piecewise-structured signals (via non-uniform interval partitioning with change-points), multiple levels of resolution and noise, a technical validation suite, and publicly available Python code to facilitate use. CoSiBD has been previously used in research presented at the IEEE COINS 2024 conference^10^, with good preliminary results for signal reconstruction using deep learning. CoSiBD is made available to support further development in deep learning approaches for temporal super-resolution.

To further position CoSiBD with respect to existing public synthetic time-series resources, we summarize in the next subsection representative datasets and simulators and highlight the practical gap addressed by our approach.

### Related synthetic time-series resources

Publicly available synthetic resources for temporal signals exist, but they are typically designed for tasks other than time-series SR, or they target a specific domain. In wireless communications, the RadioML family provides large collections of synthetic complex I/Q sequences with varying SNR (Signal-to-noise ratio) and channel impairments, mainly to benchmark automatic modulation classification rather than paired SR reconstruction^11–13^. In biomedical signal processing, physiological simulators such as ECGSYN (electrocardiography) and SEREEGA (EEG) enable controlled generation with tunable morphology, sampling settings, and noise, supporting method development when real data access is constrained^14–16^. In power systems, LoadGAN provides multi-resolution generation of load time series across sampling rates and time horizons (from sub-second to long-term scales), but it is not distributed as a standardized paired SR benchmark^17^. Domain-specific paired low-/high-resolution training data can also be produced via physical forward modeling, e.g., low- and high-resolution 1D seismic traces for learning-based resolution enhancement^18^.

Table 1 provides a concise view of representative public synthetic resources for temporal signals and clarifies how they relate to the specific needs of time-series super-resolution (SR). We summarize each resource by its target *Domain* and *Form* (fixed dataset vs. simulator/generator), and we highlight practical properties that determine whether a resource can be used as an SR benchmark: whether it provides *paired LR–HR data*, whether it supports *multi-resolution* generation, what *noise/artifact* mechanisms are available, and the *reproducibility granularity* (dataset-level variability vs. per-signal deterministic regeneration).

**Table 1:** Representative publicly available synthetic time-series datasets and simulators related to signal processing and learning.

Resource	Domain	Form	Paired LR–HR SR	Multi-resolution	Noise / artifacts	Reproducibility granularity
**CoSiBD (this work)**	Generic time series (complex-structured signals)	Dataset + generator	Yes (LR → HR targets)	Yes (150/250/500/1000 → 5000)	Gaussian + structured interference; primary benchmark uses direct decimation	Per-signal metadata; deterministic regeneration (seed-controlled)
RadioML 2016.10A^11,12^	Wireless communications (I/Q)	Dataset	No (classification benchmark)	N/A (not SR)	Variable SNR + channel impairments	Dataset-level (labels/SNR); not per-sample “recipe”
RadioML 2018.01A^13^	Wireless communications (I/Q)	Dataset	No (classification benchmark)	N/A (not SR)	Simulated channel effects + SNR variability	Dataset-level; not SR-paired
ECGSYN^14,15^	ECG (physiology)	Simulator/tool	Configurable	Configurable (via sampling settings)	Model-based; supports controlled variability	Configurable via simulator parameters (user-defined)
SEREEGA^16^	EEG (physiology)	Simulator/toolbox	Configurable	Configurable (user-defined)	Supports noise and event-related components	Configurable via simulator parameters (user-defined)
LoadGAN^17^	Power systems load time series	Generator/tool	No (generation)	Yes (variable sampling rates)	Domain-specific variability (load patterns)	Tool-based; generation is configurable
Synthetic LR–HR seismic traces (example)^18^	Seismic traces (geophysics)	Paper-specific paired data	Yes (LR–HR pairs)	Typically limited (study-specific)	Study-dependent	Paired data available for the study; limited generality

Overall, existing resources offer important strengths but also exhibit limitations when viewed through an SR-benchmark lens. Domain-specific datasets such as RadioML provide large-scale controlled variability (e.g., SNR and channel impairments) and are highly valuable for tasks like modulation classification, but they are not distributed as paired LR–HR SR targets. On the other hand, physiological simulators (e.g., ECGSYN and SEREEGA) enable flexible generation with tunable morphology, sampling settings, and noise, which in principle can be used to create LR–HR pairs; however, they typically do not provide a standardized, fixed paired benchmark designed explicitly for SR evaluation across multiple difficulty levels. Similarly, multi-resolution generators in other domains may capture realistic structure at multiple sampling rates, but often lack an aligned, reproducible LR–HR pairing protocol and a unified dataset release that facilitates direct, comparable benchmarking across methods.

These observations motivate the need for a public resource that combines: (i) fixed, aligned LR–HR pairs for SR, (ii) controllable nuisance modeling (noise and structured interference), and (iii) strong reproducibility at the per-signal level.

CoSiBD is designed to close the gap by providing a fixed, standardized set of LR–HR pairs with explicit nuisance modeling (noise and structured interference) and comprehensive metadata, enabling reproducible benchmarking across multiple difficulty levels (defined by varying downsampling factors and noise intensities).

## Methods

**Fig. 1:**
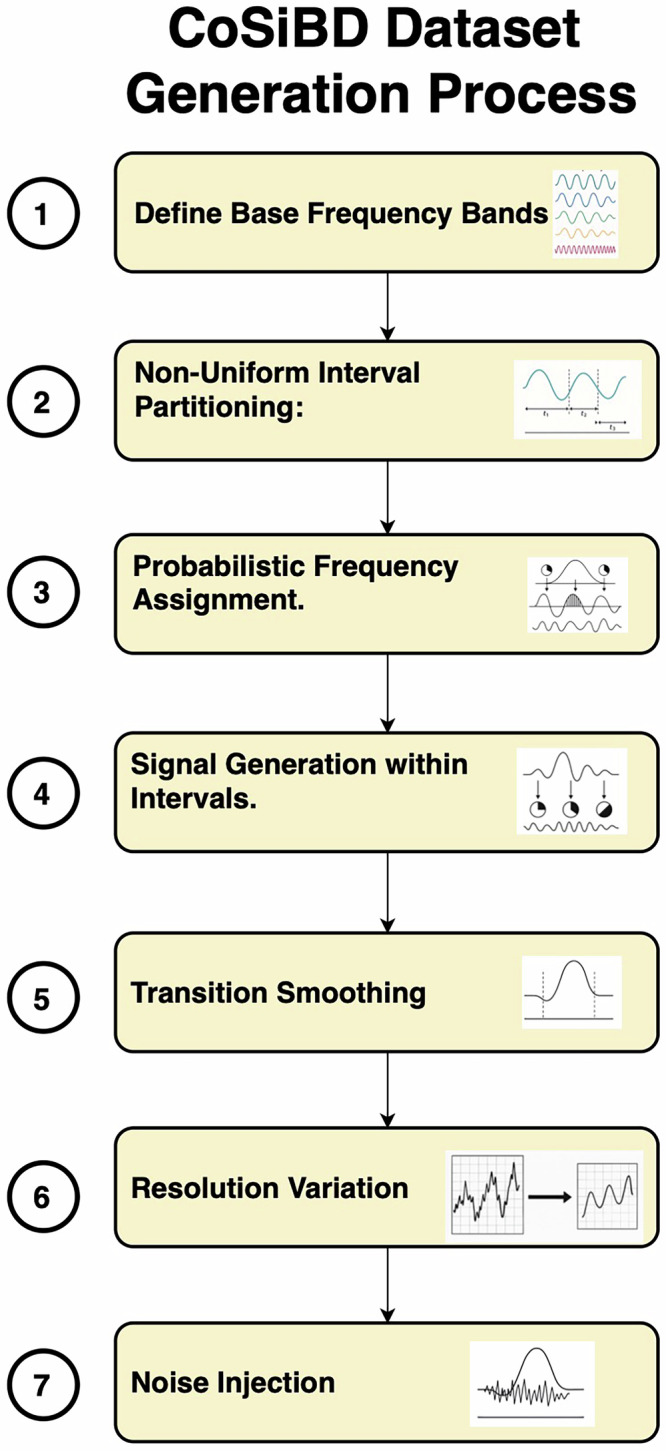
Schematic overview of the CoSiBD signal generation process.

The methodology used to generate the synthetic temporal signals that constitute the CoSiBD dataset is illustrated in Fig. 1, and will be explained later. The signal generation process is designed to produce time series exhibiting structural properties such as variable frequency content, smooth transitions, and intermittent high-frequency activity. A key aspect of the procedure is the generation of signals at multiple temporal resolutions, enabling the construction of paired datasets for super-resolution (SR) benchmarking.

### Design rationale

**Fig. 2:**
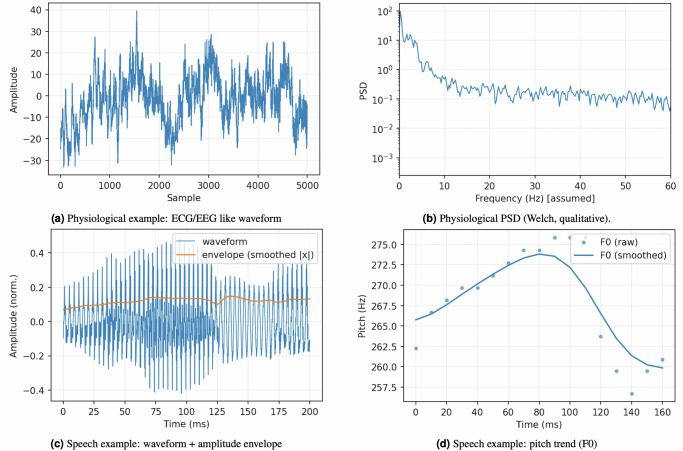
Examples of qualitative properties motivating the CoSiBD design, specifically non-stationarity and spectral structure (**a**,**b**), and envelope-pitch dynamics (**c**,**d**).

We have designed our dataset so it exhibits (i) non-stationary regime changes, (ii) coexisting low- and high-frequency components with intermittent transients, (iii) smooth amplitude-envelope evolution, and (iv) slow baseline drift and measurement noise. CoSiBD instantiates these properties via non-uniform interval partitioning with change-points, separate low/high-frequency bands, spline-based envelopes and frequency profiles, and explicit offset/noise terms. Figure 2 provides qualitative examples of these motivating properties found in time series.

The design of CoSiBD is informed by the inherent limitations of standard synthetic datasets in capturing the irregular morphologies of time series. As shown in Fig. 2 (panels a–b), in many scenarios signals are rarely stationary, often exhibiting abrupt regime shifts and highly structured spectral components that pose significant challenges for temporal reconstruction. By implementing explicit change-points and multi-band frequency partitioning, CoSiBD provides a more demanding benchmark than globally stationary models.

Similarly, as shown in Fig. 2 (panels c–d), the inclusion of spline-based modulation seeks to address the continuous but non-linear fluctuations. The amplitude envelopes and F0 trajectories represent a level of temporal complexity that rigid parametric approaches often oversimplify. Using smooth spline profiles allows the framework to emulate these high-order dependencies without assuming a fixed functional form.

The signal generation pipeline involves the following steps:

- **Base frequency band definition:** A set of distinct frequency bands is defined to represent the underlying spectral content of the signals. These can be adjusted to reflect application-specific characteristics.
- **Non-uniform interval partitioning:** The total signal duration is divided into multiple intervals of variable length. The interval lengths are determined probabilistically to introduce variability in the signal structure.
- **Frequency assignment:** Each interval is assigned a dominant frequency band, sampled according to a predefined probability distribution. This introduces spectral variation over time.
- **Signal synthesis:** A sinusoidal waveform, or a combination of sinusoids within the assigned frequency band, is generated for each interval. Signal parameters such as amplitude and phase are configurable.
- **Transition smoothing:** To avoid discontinuities at interval boundaries, a smoothing function is applied to overlapping segments. This ensures gradual transitions between intervals with different frequency content.
- **Resolution variation:** All signals are initially synthesized at a high temporal resolution (5,000 samples over the domain [0, 4*π*]). Lower-resolution versions are created using simple decimation (uniform subsampling). This keeps the SR task aligned with reconstructing the original high-resolution target; the low-resolution observation is obtained by subsampling the original sequence without pre-filtering. Reconstructing low-pass filtered signals is not an objective of CoSiBD. For reproducibility, given a high-resolution sequence *x*_HR_[*n*] of length *N* = 5000 and a target low-resolution length *M* ∈ {1000, 500, 250, 150}, we form *x*_LR_[*i*] = *x*_HR_[*n*_*i*_] using the fixed index set ${n}_{i}=\left\lfloor\frac{i\,(N-1)}{M-1}+0.5\right\rfloor$ for *i* = 0, …, *M* − 1 (applied identically to the time array). This reduces to standard stride decimation when *M* divides *N*.
- **Noise injection:** Controlled levels of synthetic noise are added to the signals. Two noise types are implemented: (1) Additive white Gaussian noise (AWGN) with configurable standard deviation (relative to signal RMS amplitude), representing broadband sensor thermal noise; and (2) structured sinusoidal noise bursts (deterministic sinusoidal components), representing narrow-band interference such as powerline hum (50/60 Hz). Noise is applied probabilistically (in the released dataset, noise_profile records p_has_noise=0.5 per signal, and the realized subset with noise is approximately half). When noise is present, the specific parameters (type, amplitude, frequency for structured noise) are recorded in the metadata, allowing users to benchmark denoising or super-resolution under specific degradation conditions.

#### Rationale for structured 50/60 Hz interference and noise

In order to emulate narrow-band interferences superimposed on broadband sensor noise, our dataset includes an optional structured sinusoidal component. This term is incorporated as a standardized benchmark for spectral disentanglement: evaluating the capacity of neural architectures to isolate target non-stationary signal dynamics from persistent periodic artifacts. While CoSiBD signals are defined over a reference domain (*τ* ∈ [0, 4*π*]), the illustrative mapping to *T* = 4*π* seconds represents 50/60 Hz powerline interference, a near-universal artifact in electronic signal acquisition. This provides a controlled scenario to quantify the robustness of super-resolution models against periodic contamination that may overlap with the signal’s baseband spectral content.

**Fig. 3:**
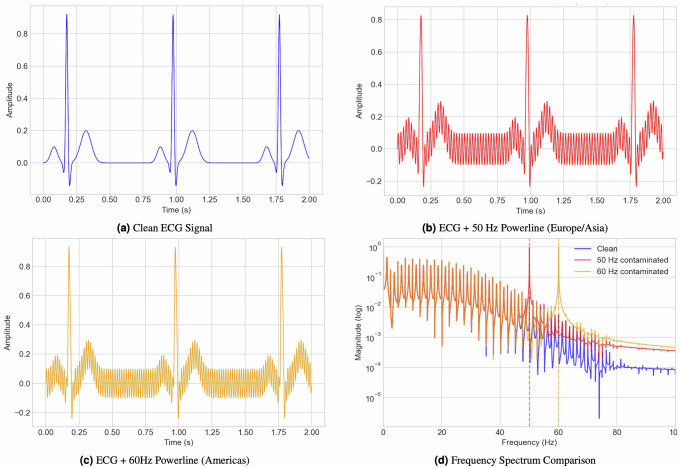
Qualitative motivation for the structured interference term used in CoSiBD.

Figure 3 illustrates this qualitative motivation. Subfigure (a) displays a clean synthetic ECG signal, serving as a baseline. Subfigure (b) shows the same signal contaminated with a 50 Hz sinusoidal component (mimicking powerline interference in Europe/Asia), while (c) illustrates the effect of a 60 Hz component (typical of the Americas). Subfigure (d) presents the frequency spectrum comparison, clearly showing the narrow-band interference spikes associated with these powerline artifacts. This explicitly models periodic contamination^19^, with the purpose of including nuisance factors that SR models must handle.

#### Sampling units and frequency interpretation

CoSiBD signals are provided as discrete sequences *x*[*n*] (e.g., *N* = 5, 000 samples) that are directly used as inputs/targets by SR models. The internal generation domain *τ* ∈ [0, 4*π*] is a reference parameterization; interpreting it as physical time requires choosing a duration *T* (in seconds) for the reference interval. Under this convention, the implied sampling rate is *f*_*s*_ = *N*/*T* and all frequencies reported in Hz scale linearly with 4*π*/*T*. Throughout this manuscript, when reporting example frequencies in Hz we adopt the illustrative convention *T* = 4*π* s, yielding *f*_*s*_ ≈ 5000/(4*π*) ≈ 398 Hz; other equally valid mappings exist depending on application. Consequently, any band-specific interpretation in Hz (e.g., “low/high” frequency ranges) should be understood under the chosen *T*; changing *T* rescales all reported Hz values while preserving the underlying discrete sequences, which is a key feature of CoSiBD’s design.

**Fig. 4:**
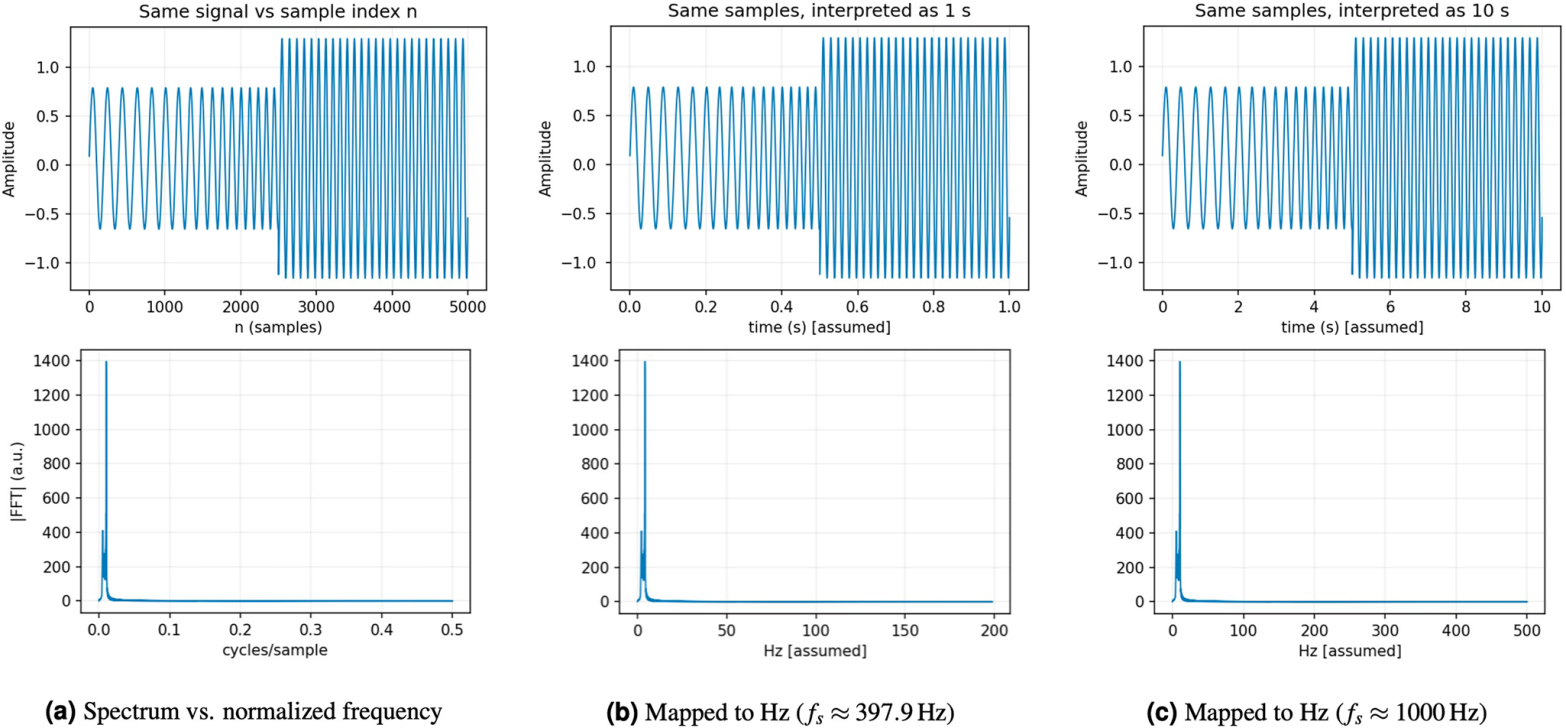
Frequency domain mapping under different sampling rate assumptions.

Figure 4 clarifies this convention using three panels (a–c). Panel (a) shows the normalized frequency spectrum (cycles/sample), which is intrinsic to the discrete sequence. Panels (b) and (c) illustrate how this same spectrum maps to physical units (Hz) under different assumed sampling rates: (b) assumes *f*_*s*_ ≈ 397.9 Hz (corresponding to *T* = 4*π* s), while (c) assumes *f*_*s*_ ≈ 1000 Hz. This demonstrates that while the discrete data remains identical, the physical interpretation scales with the user-defined duration.

## Data Records

The Complex Signal Benchmark Dataset (CoSiBD) is publicly available on Zenodo^20^ and consists of synthetic temporal signals, mainly created to support the development and evaluation of temporal super-resolution (SR) algorithms, and also to train deep learning models that can be used for SR. This section provides an overview of the dataset structure, content, and storage format, as well as the parameters that rule the generation of data and the metadata that enrich our dataset.

The dataset comprises 2,500 high-resolution signals, each with corresponding subsampled versions at four resolution levels, organized into three main categories:

- **High-resolution signals**: 2,500 signals with 5,000 samples each, spanning the domain *T* = [0, 4*π*] (s), which, under the illustrative convention used, corresponds to *f*_*s*_ = 5000/(4*π*) ≈ 398 Hz. Each signal is stored in three formats: NumPy compressed format (.npz), plain text (.txt), and JSON (.json). Per-signal metadata (frequency profiles with explicit change-points (base_points and high_freq_points) and segment labels (variation_type), amplitude envelopes, spline parameters, vertical offsets, noise configurations, and seeds) is provided in a consolidated JSON file (signals_metadata.json) with one entry per signal, enabling exact regeneration.
- **Simple subsampled signals**: Uniform decimation (uniform subsampling) of each signal to four target resolutions: 150 (illustrative *f*_*s*_ ≈ 11.9 Hz for *T* = 4*π* s), 250 (illustrative *f*_*s*_ ≈ 19.9 Hz for *T* = 4*π* s), 500 (illustrative *f*_*s*_ ≈ 39.8 Hz for *T* = 4*π* s), and 1,000 samples (illustrative *f*_*s*_ ≈ 79.6 Hz for *T* = 4*π* s). These low-resolution versions serve as inputs for SR benchmarking against the original 5,000-sample target. Stored in .npz, .txt, and .json formats. CoSiBD intentionally bypasses anti-aliasing filtration prior to decimation. This design choice establishes the dataset as a benchmark for evaluating the capacity of neural architectures to resolve spectral ambiguities and reconstruct high-frequency information from raw, aliased discrete sequences, rather than from pre-filtered, band-limited signals.

The dataset is provided as consolidated files under SignalBuilderC/data/. High-resolution signals are stored as signals_ high_ resolution_ 5000.[npz∣txt∣json]. Simple subsampled (decimated) signals are stored as signals_ subsampled_ simple_ {150,250,500,1000}.[npz∣txt∣json]. Dataset-level metadata (described later in detail) and configuration are stored in signals_ metadata.json (per-signal metadata, one entry per signal), signals_ metadata_ consolidated_ 2500.json, and dataset_ summary.json.

Regarding the three formats used for both high-resolution and subsampled signals, we provide here some additional information for each format: (1) NumPy compressed format (.npz) containing the signal array, time array, and (for high-resolution only) clean signal without noise; (2) consolidated plain text format (.txt) with one signal per row (samples separated by whitespace) for maximum portability; and (3) JSON format (.json) with both time and signal arrays for web-based applications and interoperability.

Reproducibility is ensured through documented seeds: each high-resolution signal is generated using a unique seed (ranging from 10,000 to 12,499), enabling exact regeneration of individual signals or the entire dataset. All generation parameters (described later in detail) are stored in metadata JSON files, including: (1) frequency profile parameters—tau_frequency values from uniform distribution [1, 2] with 0.05 step; (2) amplitude envelope parameters—amplitude_spline_type is predominantly zero_order (approximately 70%), otherwise tension with tau_amplitude drawn from {1, 3, 5, 8, 10, 12, 15, 20}; (3) vertical offsets—normally distributed (mean=0, SD=3.0); and (4) noise configurations—per-signal noise_profile with p_has_noise=0.5 (and p_gaussian=0.5 when noise is present).

### Metadata schema and example

CoSiBD provides per-signal metadata to support (i) deterministic regeneration, (ii) principled partitioning (e.g., by noise type/level or segment labels), and (iii) analysis of the piecewise structure induced by change-points. Table 2 summarizes representative fields contained in signals_metadata.json. A minimal example entry is shown below (one signal; values truncated for brevity).

**Table 2:** Metadata schema describing the JSON structure for each signal.

Field	Type	Example	Meaning
signal_id	String	“signal_0000”	Unique identifier for the generated signal.
index	Integer	0	Numeric index of the signal (0 to N-1).
seed	Integer	10000	Seed used for deterministic generation of this specific signal.
t_start, t_end	Float	0.0, 12.56	Start and end time of the signal domain.
base_points	Array	[[0.0, 2.07]...]	Control points (*t*, *f*) for the frequency spline.
high_freq_points	Array	[[0.0, 0.0]...]	Control points (*t*, *f*) for an optional high-frequency component.
variation_type	List[Str]	[“low”, “high”, “high”]	Categorical labels aligned with base_points anchors (same length), describing the intended band at each change-point (e.g., “low”, “high”, “no_change”); interval labels are derived between successive anchors.
tau_frequency	Float	1.15	Tension parameter for the frequency spline.
amplitude_spline_type	String	“zero_order”	Interpolation family for the amplitude envelope (e.g., step-wise or tension spline).
tau_amplitude	Float / String	10.0	Tension parameter for the amplitude spline (or “N/A” for zero-order).
amp_knots	Array	[0.0, 6.28, 12.56]	Knot times defining the amplitude envelope segments.
amp_values	Array	[0.72, 1.22, 0.96]	Amplitude values at knots (piecewise-constant or spline-interpolated).
noise_profile	Dict	{...}	Parameters for added noise (if any).

Key fields define the frequency trajectory (base_points, variation_type) and amplitude envelope, allowing precise reconstruction or filtering.

**Fig. 5:**
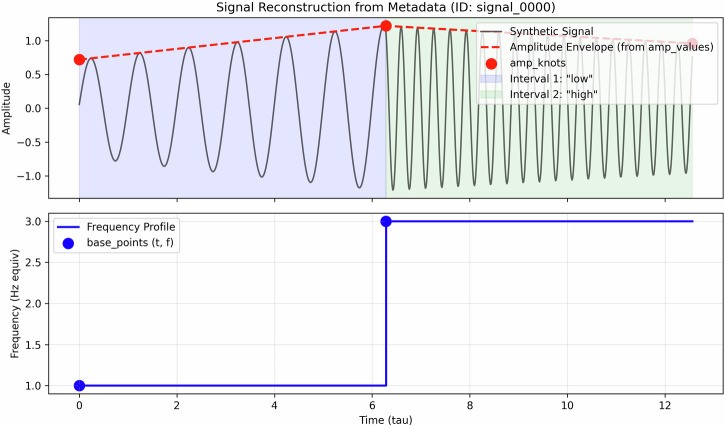
Visual representation of an illustrative metadata record for signal_0000 (Example 1). The plot illustrates how base_points define temporal intervals and frequency targets, while amp_values control the amplitude envelope segments, corresponding directly to the JSON structure.

Table 2 details the key fields describing each signal, specifically the variation_type list which provides categorical labels aligned with the base_points anchors (e.g., “low”, “high”, or “no_change”), enabling users to filter for signals containing specific dynamic behaviors. The following JSON snippet shows a simplified illustrative metadata record (Example 1; values truncated for readability). Figure 5 provides a visual counterpart of the same record: it highlights how consecutive base_points (and, when present, high_freq_points) define temporal intervals and frequency targets, while variation_type provides the corresponding per-anchor labels (here, yielding two intervals: “low” then “high”), and amp_knots/amp_values define the amplitude envelope.

{“t_start”: 0.0,“t_end”: 12.566370614359172,“fs_high”: 397.88735772973837,“tau_frequency”: 1.15,“amplitude_spline_type”: “zero_order”,“tau_amplitude”: “N/A”,“vertical_offset”: 0.06905161748158965,“base_points”: [[0.0, 1.0], [6.28, 3.0], [12.56, 3.0]],“high_freq_points”: [[0.0, 0.0], [6.28, 0.0], [12.56, 0.0]],“variation_type”: [“low”, “high”, “high”],“amp_knots”: [0.0, 6.28, 12.56],“amp_values”: [0.72, 1.22, 0.96],“noise_profile”: “has_noise”: true, “noise_type”: “gaussian”, “p_has_noise”: 0.5, ...,“seed”: 10000,“signal_id”: “signal_0000”,“index”: 0}

### Parameters for signal generation

As we anticipated before, our dataset is generated from a set of configuration parameters summarized in Table 3. Each high-resolution signal was generated with a unique seed (10,000–12,499) and sampled parameter values within the defined ranges, supporting diversity while maintaining reproducibility. Parameters marked as *Sampled (seed-controlled)* are drawn per-signal using the recorded seed, while fixed defaults are held constant across the dataset. Unless otherwise stated, these defaults correspond to the settings used in the Zenodo release, and the CLI follows the same behavior unless a user explicitly overrides a parameter.

**Table 3:** Configuration parameters used to generate the dataset.

Parameter	Range / Options	Default	Description
Low Frequency	1–5 Hz	Sampled (seed-controlled)	Low-frequency component (*T* = 4*π* s convention).
High Frequency	20–100 Hz	Sampled (seed-controlled)	High-frequency variations for transitions.
Change Points	2–11	Sampled (seed-controlled)	Number of frequency transitions per signal.
Change Locations	Continuous	Sampled (seed-controlled)	Time locations where transitions occur.
Variation Type	{low, high, no_change}	Balanced	Category of frequency change per segment.
Amplitude Range	3–16	Sampled (seed-controlled)	Bounds for amplitude envelope generation.
Vertical Offset	*N*(0, 3.0)	0.0	Normally distributed offset added to signals.
Spline Type	Zero-Order (70%), Tension (30%)	—	Interpolation method for envelopes.
Tension (freq)	[1, 2]	1.5	Tension parameter for frequency splines.
Tension (amp)	{1, 3, 5, 8, 10, 12, 15, 20}	Sampled (seed-controlled)	Tension parameter for amplitude splines ("N/A" for zero-order).
Noise Prob.	0.0–1.0	0.5	Probability of adding noise to a signal.
Seed	Integer	—	Unique initialization seed per signal.

Ranges define the sampling space for the 2,500 signals, ensuring diversity. When not explicitly set by the user, parameters are either sampled per signal (seed-controlled) or take fixed defaults as listed (defaults used for the Zenodo release).

Concretely, generation proceeds as a deterministic pipeline conditioned on the seed: (i) the seed initializes all pseudo-random draws; (ii) a set of frequency-trajectory and envelope parameters is sampled within the reported ranges; (iii) a continuous-time signal is synthesized over the fixed domain (*T* = 4*π* s convention) by combining a time-varying frequency trajectory with an amplitude envelope; and (iv) optional nuisance terms (offset and additive noise) are applied. Therefore, fixing the seed fully determines the sampled parameters and the resulting signal instance, which supports exact reproduction and controlled ablations.

#### Frequency trajectory

The overall (instantaneous) frequency evolution is controlled by the sampled low/high frequency bands (“Low Frequency” and “High Frequency”), the number of transitions (“Change Points”), and their temporal placement (“Change Locations”). The categorical “Variation Type” ({low, high, no_change}) provides labels aligned with the base_points anchors, indicating whether the trajectory targets the low band, the high band, or remains stable around each change-point; this supports controlled balancing and downstream filtering via metadata. The smoothness/shape of the resulting trajectory is governed by the spline settings, including tau_frequency (“Tension (freq)”).

#### Amplitude envelope

Independently of the frequency trajectory, the signal amplitude is modulated by an envelope whose overall scale is constrained by “Amplitude Range”. The envelope shape is generated via the specified interpolation scheme (“Spline Type”) and, when applicable, by the sampled “Tension (amp)” parameter. This yields signals with comparable frequency content but different amplitude dynamics.

#### Offsets and noise

To emulate common acquisition artifacts without tying the generator to a specific instrument, we add a per-signal “Vertical Offset” and inject additive noise stochastically according to “Noise Prob.”. When noise is enabled for a given signal, its parameters are recorded in metadata to preserve reproducibility.

#### Resolution and sampling

**Fig. 6:**
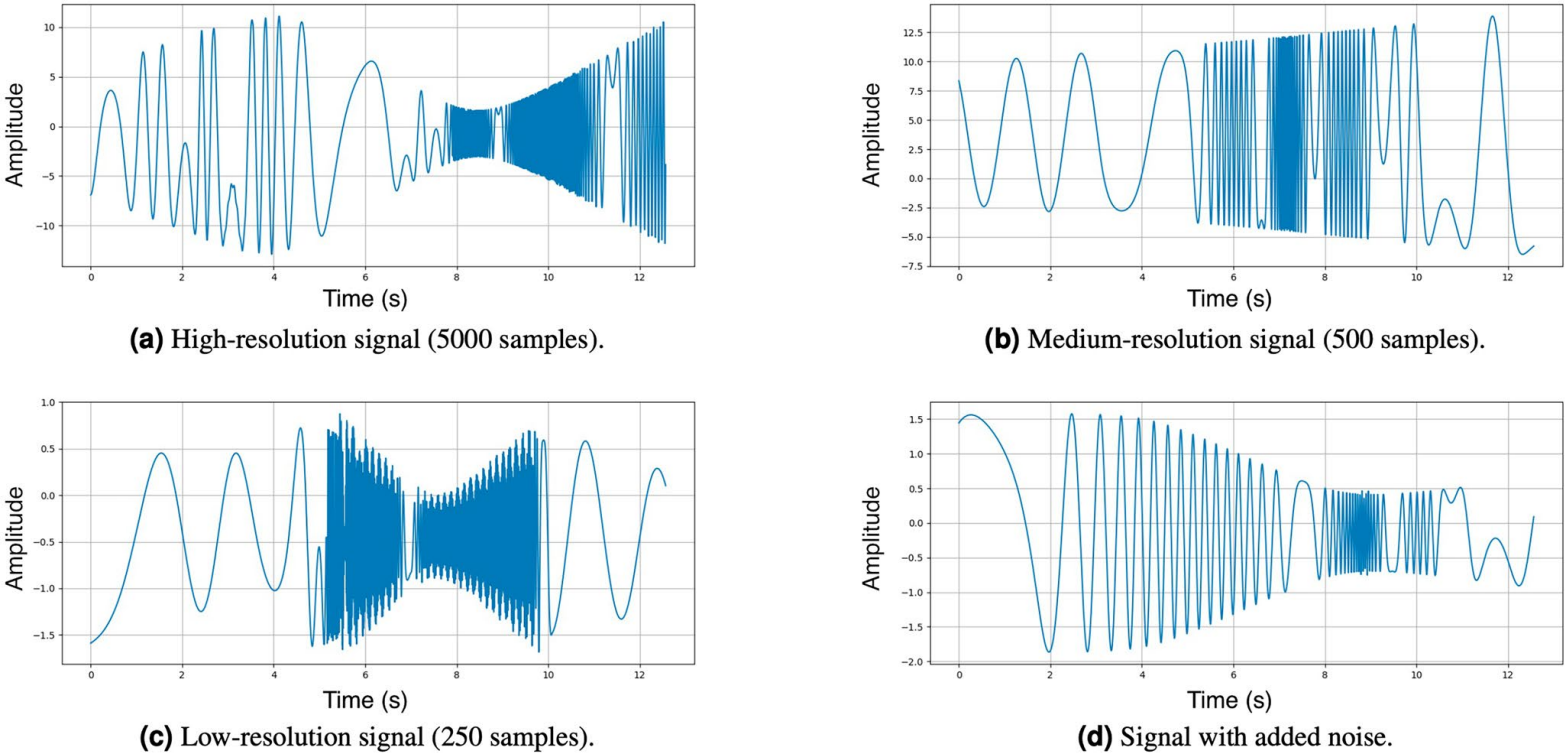
A synthetic signal sampled at different resolutions: (**a**) high (5000 samples), (**b**) medium (500 samples), (**c**) low (250 samples), and (**d**) with added noise. These examples reflect the multi-resolution and noise conditions present in the dataset.

Each instance is first generated at the target high-resolution length (e.g., 5,000 samples in the released dataset), and lower-resolution views are derived by subsampling, enabling consistent multi-scale analysis and SR benchmarking (Fig. 6).

#### Mathematical formulation

Let *t* ∈ [*t*_start_, *t*_end_] with the paper’s convention *T* = *t*_end_ − *t*_start_ = 4*π* s. A generated signal can be written as

1 $$x(t)=v+a(t)\,\sin \,\left(\phi (t)\right)+\varepsilon (t),$$

where *v* is a vertical offset, *a*(*t*) ≥ 0 is the amplitude envelope, and *ε*(*t*) is an optional additive noise term. The instantaneous frequency trajectory is encoded through the phase derivative

2 $$f(t)=\frac{1}{2\pi }\,\frac{d\phi (t)}{dt},$$

and is constructed from a spline defined by a set of control points and a tension parameter (Table 3). This separation (frequency trajectory vs. amplitude envelope) allows generating signals that share the same transition structure but differ in amplitude dynamics, or vice versa.

#### Mapping Table 3 to metadata fields

The parameters in Table 3 correspond to concrete entries in each per-signal metadata record (Fig. 5 shows an illustrative example):

- *Seed*  → seed (and derived identifiers signal_id, index). Fixing seed reproduces all sampled values.
- *DomainT* = 4*π* convention → t_start, t_end.
- *Change points / locations / variation type*  → base_points (frequency control points), variation_type (interval labels), and auxiliary arrays such as high_freq_points used to represent high-frequency targets during transitions.
- *Frequency tension*  → tau_frequency.
- *Amplitude range / spline settings*  → amplitude_spline_type, tau_amplitude, and the envelope knot representation amp_knots and amp_values.
- *Vertical offset*  → vertical_offset.
- *Noise probability and settings*  → noise_profile (including whether noise was applied and the sampled noise parameters for that signal).

Some fields (e.g., fs_high) are reported as part of the complete record to make downstream processing explicit; they are derived from the chosen resolution and the domain convention.

CoSiBD spans multiple controlled axes of variation (Table 3), including the number and location of change points, categorical transition types, low/high frequency bands, and amplitude-envelope configurations. These controlled variations provide reproducible coverage of phenomena such as non-stationarity, transient high-frequency events, and additive noise.

**Fig. 7:**
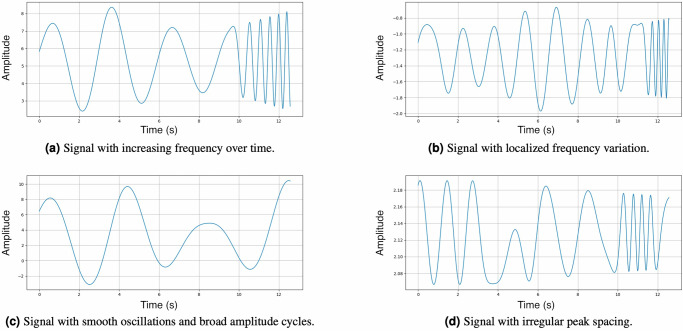
Examples of synthetic signals in the dataset generated with different parameter configurations. Each signal presents a distinct temporal profile.

Figure 6 shows a representative signal from the dataset sampled at different resolution levels, as well as a version with added noise. This illustrates the variety of sampling and noise conditions included in CoSiBD. Figure 7 displays four additional synthetic signals generated using different configuration parameters. These examples demonstrate the variability in temporal structure across instances in the dataset.

### Custom Dataset Generation

**Fig. 8:**
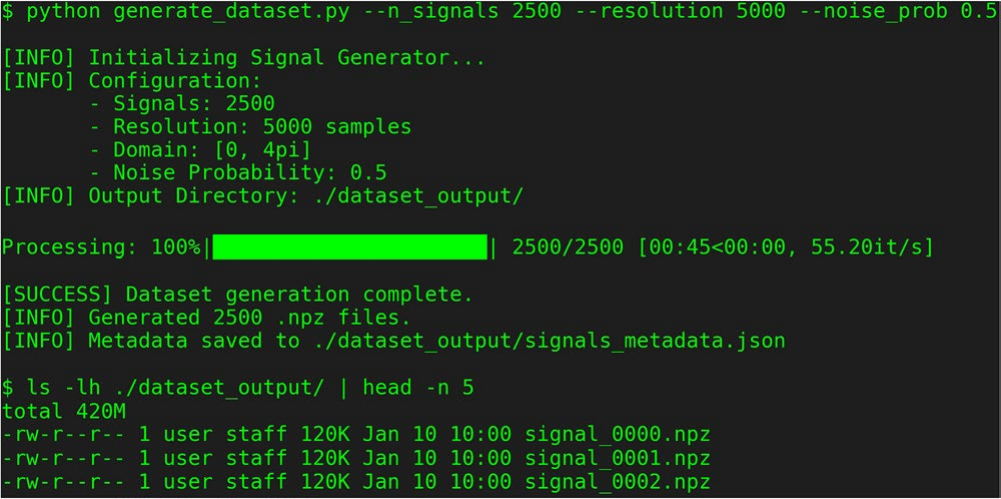
Demonstration of the CoSiBD Command Line Interface (CLI). Users can generate new dataset instances by specifying parameters such as signal count, sampling resolution, and noise probability directly from the terminal.

In addition to the pre-generated dataset, the CoSiBD package includes a command-line interface (CLI) that allows users to generate custom datasets with their own parameter distributions. Figure 8 demonstrates the usage of this tool. The command specifies the number of signals (–n_signals), the target high-resolution length in samples (–resolution, e.g., 5000), and the probability of applying noise to each signal (–noise_prob, e.g., 0.5, meaning noise is injected into roughly half of the signals). The output log confirms the resolved configuration (signal count, domain, and output directory), reports progress during generation, and summarizes the created artifacts (per-signal files and the consolidated signals_metadata.json).

## Technical Validation

This section evaluates the signal generation procedure by analyzing spectral properties under different conditions, including the distribution of dominant frequencies, spectral stability across sampling rates, and the effect of noise. Additionally, we provide a multi-scale super-resolution benchmark to demonstrate the dataset’s utility in training deep learning models, and illustrative transfer learning experiments on EEG and speech data. These analyses aim to assess variability and stability under the reported settings, and to document the dataset’s behavior for reproducible use. Below, the methodologies and results are described in detail.

### Validation Context

Experimental parameters were selected to support reproducibility and to illustrate representative behaviors of the generator under the reported settings. The number of signals (*n* = 50 for spectral analysis, *n* = 2500 for benchmarks) provides a compact but informative sample to summarize variability. Sampling resolutions (ranging from 150 to 5000 samples) reflect scenarios requiring different levels of detail, aligning with typical signal processing use cases. Noise amplitudes (Gaussian noise with *σ* ∈ [0.0, 0.2]) were motivated by common acquisition artifacts, with the goal of providing a controllable benchmark rather than an exhaustive model of any specific measurement pipeline. Unless otherwise stated, signal-generation settings follow the configuration in Table 3.

### Analysis of Dominant Frequency Distribution

To assess the stability and variability of the primary spectral components, we analyzed the distribution of dominant frequencies across multiple generated signals. A total of fifty independent signals were synthesized using identical input parameters. To examine their spectral characteristics, we computed the power spectral density (PSD) of each signal, which quantifies how signal power is distributed across different frequencies.

The PSD was estimated using Welch’s method, selected for its ability to reduce noise and provide a smoother spectral representation^21^. This method stabilizes spectral estimation by dividing the signal into overlapping segments, computing their individual spectra, and averaging them. This reduces variance from random fluctuations and yields a smoother estimate. For each signal, the dominant frequency was identified as the frequency at which the PSD reaches its maximum value. This corresponds to the most prominent spectral component, indicating where the signal concentrates most of its energy.

By analyzing the distribution of dominant frequencies across the dataset, we evaluate whether the generated signals exhibit consistent spectral patterns or if there is significant variation. High consistency would indicate stability in the data generation process, whereas high variability could suggest the influence of stochastic sampling effects (seed-controlled).

**Fig. 9:**
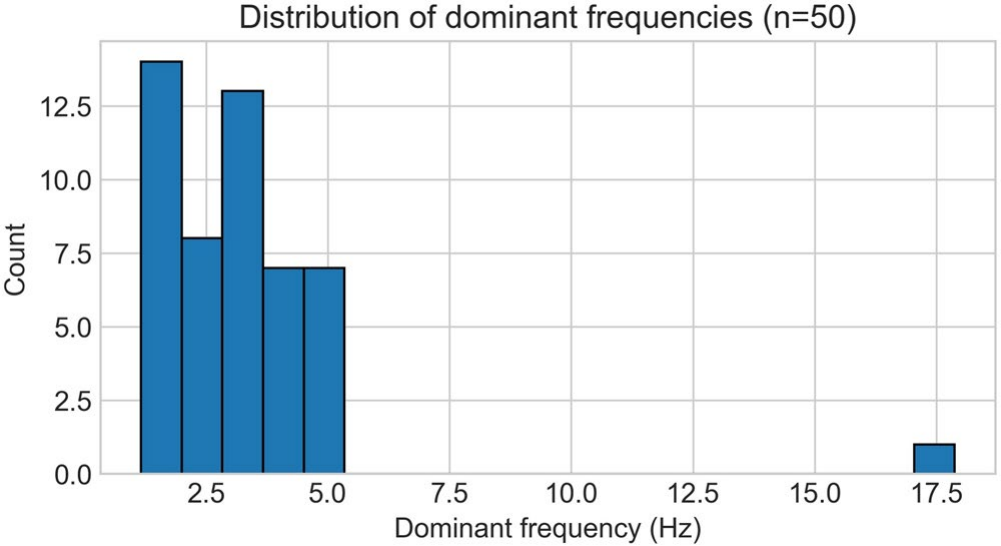
Distribution of dominant frequencies in 50 independently generated signals (reported in Hz, assuming the illustrative convention *T* = 4*π* s; for other time domains, the axis rescales by 4*π*/*T*).

The results, shown in Fig. 9 and Table 4, indicate that the dominant frequency (reported in Hz under the illustrative convention *T* = 4*π* s) is predominantly low under the reported settings. Quantitatively, the mean dominant frequency is 3.428 Hz with a standard deviation of 2.567 Hz, while the observed range spans 1.166–17.877 Hz (Table 4). Figure 9 shows a strong concentration in the low-frequency region, with a thinner tail toward higher values, corresponding to a smaller subset of realizations where the strongest spectral peak shifts upward. This pattern is consistent with the stochastic sampling of frequency profiles (e.g., random control points and segment-wise variations): signals share the same parameter ranges, but some draws produce higher instantaneous-frequency segments that become dominant in the PSD. Overall, the concentration around low frequencies combined with controlled spread supports the goal of stable primary structure with deliberate diversity, which is desirable for training models that must generalize across plausible spectral configurations^22^.

**Table 4:** Summary statistics of dominant frequencies, including average, standard deviation, and extreme values.

Statistic	Value (Hz; illustrative *T* = 4*π* s)
Average Dominant Frequency	3.428
Standard Deviation	2.567
Minimum Dominant Frequency	1.166
Maximum Dominant Frequency	17.877

**Fig. 10:**
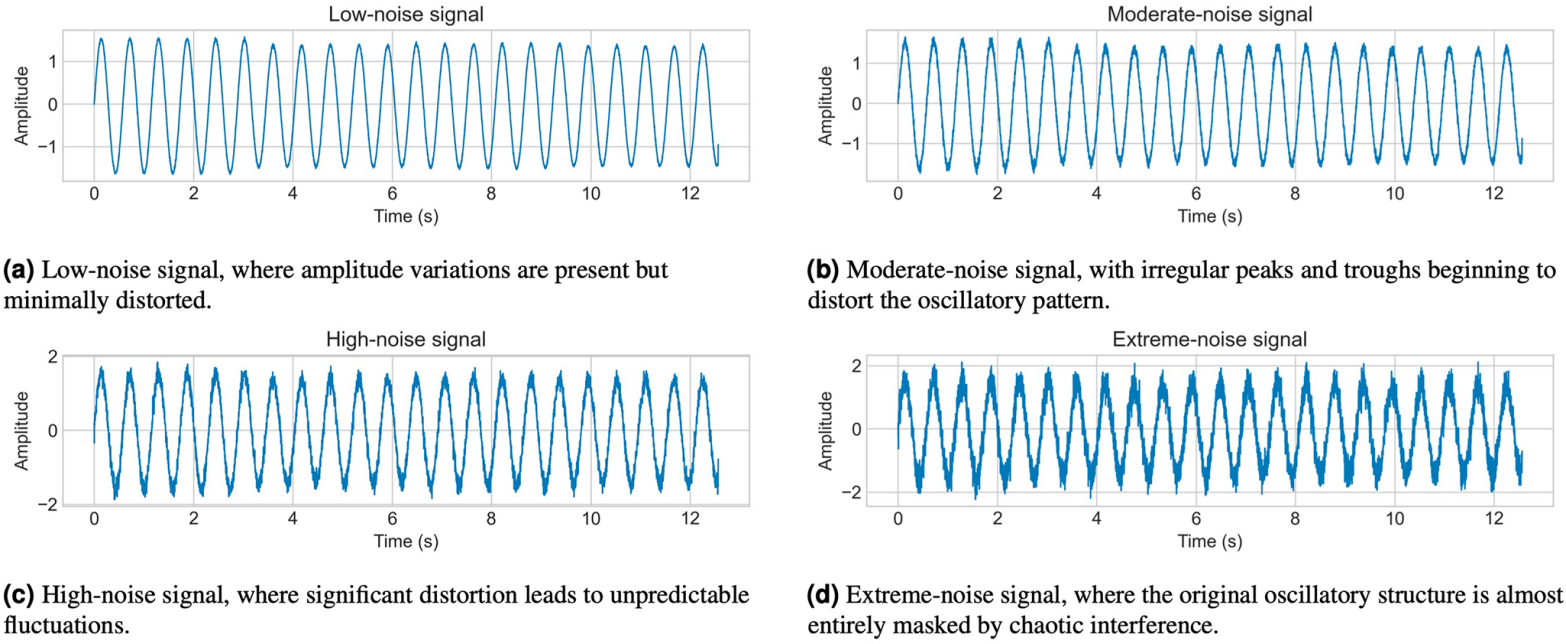
Visualization of signals under increasing noise conditions, showing how added noise progressively masks the original temporal patterns. From low (**a**) to extreme noise levels (**d**), this degradation highlights reconstruction challenges for super-resolution models.

Figure 10 presents examples of signals from the CoSiBD dataset with increasing levels of added noise, illustrating how amplitude fluctuations progressively obscure the underlying temporal structure.

### Spectral Stability Across Sampling Resolutions

This analysis aims to investigate the influence of sampling resolution (number of samples) on the robustness of spectral estimates under varying frequency content. When frequency axes are reported in Hz, they follow the illustrative convention *T* = 4*π* s; for other choices of *T*, the Hz axis rescales by 4*π*/*T*. At lower resolutions, reduced sampling density and coarser frequency grids can obscure or merge spectral peaks, compromising the ability to distinguish closely spaced spectral components^23^. Conversely, higher resolutions improve the granularity of the frequency axis, allowing for better separation of spectral features and reducing the risk of misrepresenting the signal’s underlying structure^24^.

This evaluation documents how spectral summaries vary with sampling resolution under the reported settings. The intent is to provide descriptive context for using CoSiBD at different resolutions (and computational budgets) in benchmark protocols, rather than to prescribe a universal sampling rate.

**Fig. 11:**
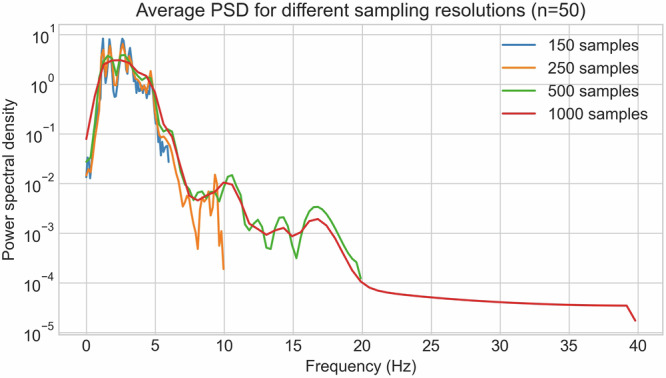
Average power spectral density (PSD) for different sampling resolutions based on 50 independent runs (Hz axis under the illustrative convention *T* = 4*π* s).

As shown in Fig. 11, lower sampling resolutions, specifically the blue curve (150 samples) and the orange curve (250 samples), exhibit a noticeable reduction in detail within the higher-frequency range. These lower-resolution curves display greater fluctuations, consistent with the theoretical effects of subsampling and aliasing^25^. In contrast, the higher sampling resolutions (500, 1000 samples) demonstrate a smoother and more stable spectral profile. This analysis confirms that while lower sampling rates introduce aliasing artifacts, the dat

aset provides spectral fidelity comparable to theoretical expectations when sufficient resolution is employed.

### Impact of Noise on Frequency Characteristics

**Fig. 12:**
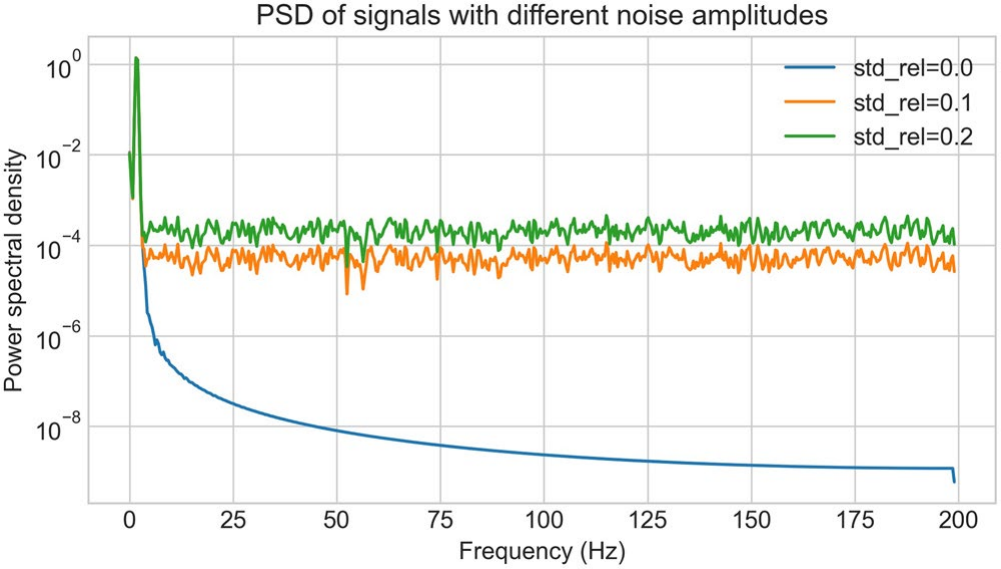
Power spectral density (PSD) of signals generated with different noise amplitudes (Hz axis under the illustrative convention *T* = 4*π* s).

We analyze how varying the noise amplitude affects the power spectral density (PSD), with particular attention to differences between low- and high-frequency regions. Figure 12 illustrates this effect under the reported settings.

Across these settings, as the noise amplitude increases—from 0.0 (blue curve) to 0.2 (green curve)—the estimated PSD exhibits increased variability at higher frequencies, while the low-frequency region remains comparatively stable. This stability suggests that CoSiBD signals retain their primary structural characteristics even under significant noise, a critical property for robust representation learning^26^.

### Multi-Scale Super-Resolution Benchmark

To illustrate a baseline use case of CoSiBD, we trained a series of convolutional neural network (CNN) models for time series super-resolution at four different scaling factors: 5× (1000 → 5000), 10× (500 → 5000), 20× (250 → 5000), and 33× (150 → 5000). All models employed the TimeSeriesSRNet architecture—a five-layer encoder-decoder network with 1D convolutional layers and bilinear upsampling, inspired by deep residual architectures for audio generation^9^. For this benchmark, the 2,500 high-resolution signals were partitioned into an experiment-specific split of 2,000 paired signals for training and 500 held-out signals for validation.

We selected this architecture as a simple 1D convolutional encoder–decoder baseline: it captures local temporal structure while providing a deterministic upsampling mechanism, enabling consistent comparisons across scaling factors. Each model was trained using mean squared error (MSE) loss, a standard objective for regression tasks requiring broad mode coverage^27^, using the Adam optimizer (learning rate 0.001). A batch size of 16 was used as a practical compromise between optimization stability and MPS memory constraints. Training was conducted on Apple Silicon GPU (MPS backend) to accelerate convergence.

**Fig. 13:**
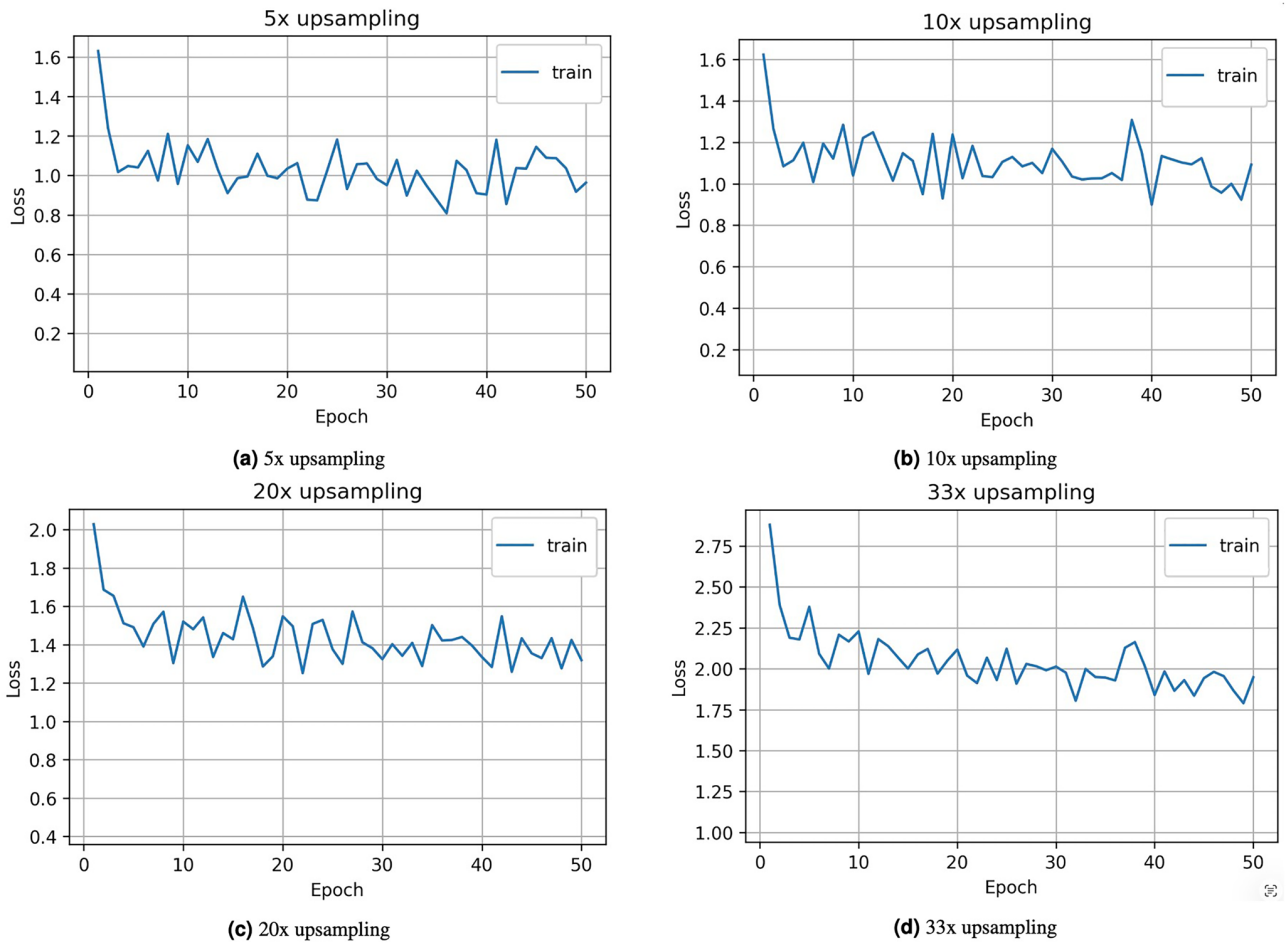
Training loss curves for each upsampling factor in the multi-scale benchmark.

Table 5 summarizes the validation performance. In these runs, validation loss increased systematically with upsampling factor, reflecting the inherent difficulty of reconstructing fine temporal details from severely undersampled inputs (Table 5, Fig. 13). Figure 13 shows the training loss trajectories separately for each scaling factor: (a) 5× (1000 → 5000), (b) 10× (500 → 5000), (c) 20× (250 → 5000), and (d) 33× (150 → 5000). In all four cases, training curves show consistent convergence without pronounced instability, indicating stable optimization under the fixed protocol.

**Table 5:** Multi-scale super-resolution benchmark results. Validation loss measured as mean squared error on 500 independent validation signals. LSD (Log Spectral Distance) quantifies spectral content deviation, while SCORR (Spectral Correlation) measures frequency-domain similarity.

Input Size	Factor	Val Loss	Epochs	Early Stop	LSD	SCORR
1000 samples	5×	0.0845	50	No	0.51 ± 0.63	0.98 ± 0.10
500 samples	10×	0.1524	50	No	0.64 ± 0.63	0.98 ± 0.10
250 samples	20×	0.4376	50	No	0.95 ± 0.67	0.98 ± 0.10
150 samples	33×	1.0326	50	No	1.21 ± 0.67	0.98 ± 0.11

All models completed the full 50-epoch training without early termination, showing stable convergence.

Across panels (a–d), the final validation loss increases as the factor grows, consistent with the expected increase in ill-posedness as fewer LR samples constrain the HR target. The 5× and 10× settings (a–b) exhibit faster convergence and lower final error, while the 20× and especially the 33× setting (c–d) show higher residual error, reflecting the greater difficulty of reconstructing fine-scale temporal detail from extreme undersampling.

**Fig. 14:**
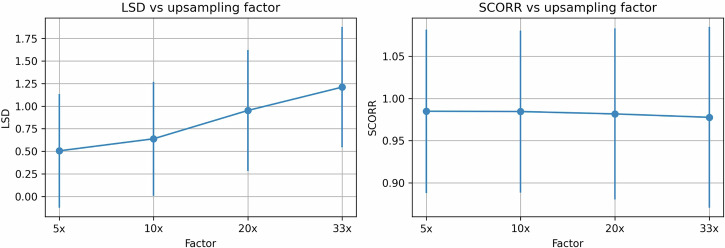
Spectral quality metrics vs upsampling factor. Left: Log Spectral Distance (LSD) increases systematically with upsampling factor, from 0.51 (5×) to 1.21 (33×). Right: Spectral Correlation (SCORR) maintains consistently high values (>0.97) across all factors. Error bars represent standard deviation over 500 validation signals per factor.

**Fig. 15:**
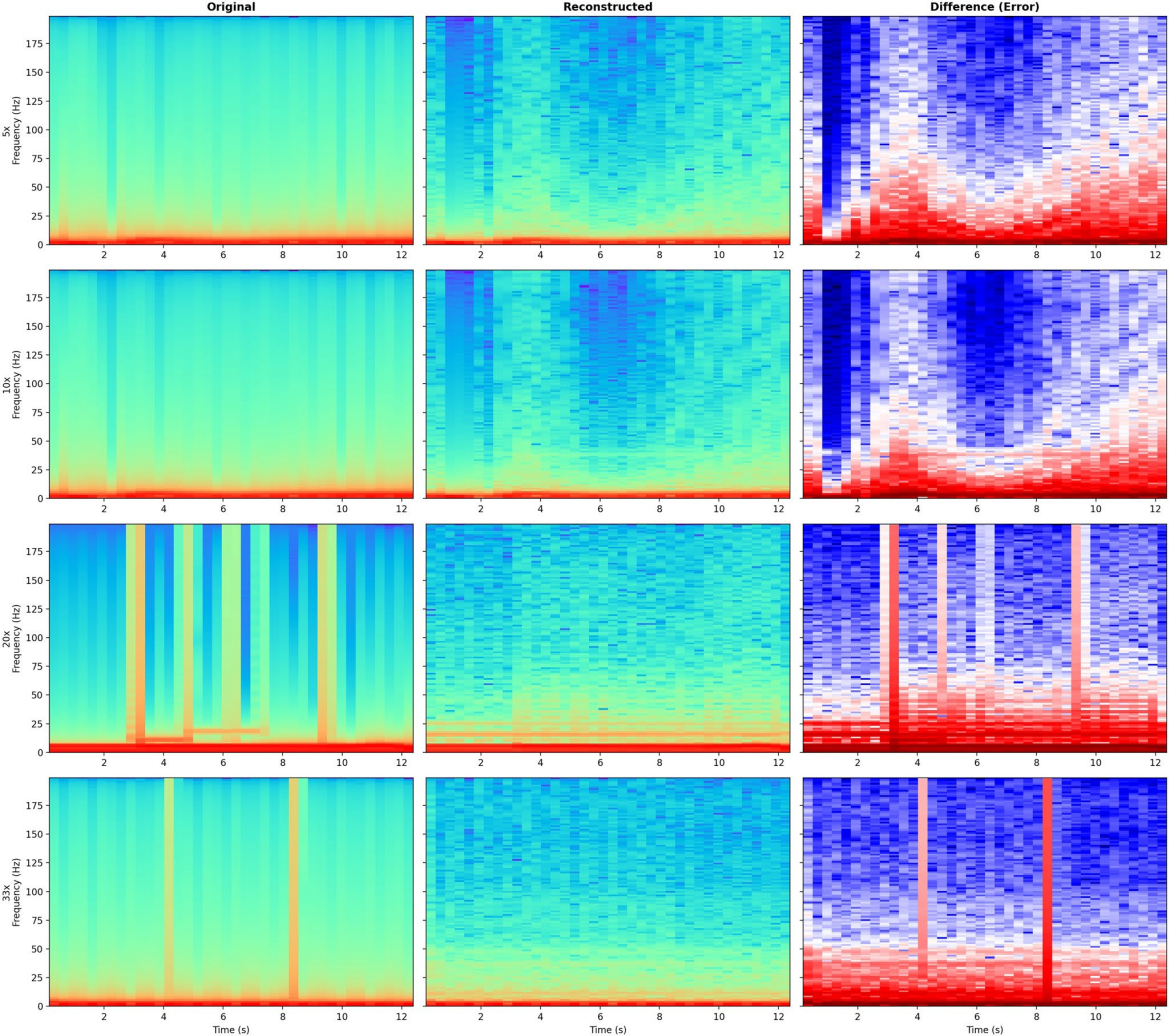
Spectrogram comparison across all upsampling factors. Each row represents a different upsampling factor (5×, 10×, 20×, 33×), showing original signal (left), CNN-reconstructed signal (center), and spectral difference (right). Reconstruction artifacts become more visible at higher upsampling rates. Representative signals selected based on median Log Spectral Distance (LSD) for each factor.

To complement amplitude-based validation, we computed spectral fidelity metrics. Log Spectral Distance (LSD) increased from 0.51 (5×) to 1.21 (33×), indicating progressively larger deviations in spectral magnitude as reconstruction becomes more challenging, while Spectral Correlation (SCORR) remained consistently high (Table 5, Fig. 14), suggesting that broad spectral structure is still largely preserved across factors^24^. Figure 15 provides representative spectrogram comparisons across all upsampling factors; the rightmost column (spectral difference) highlights where reconstruction introduces localized discrepancies (warm colors) that become more pronounced at higher factors, typically around rapid transitions and high-frequency content.

**Fig. 16:**
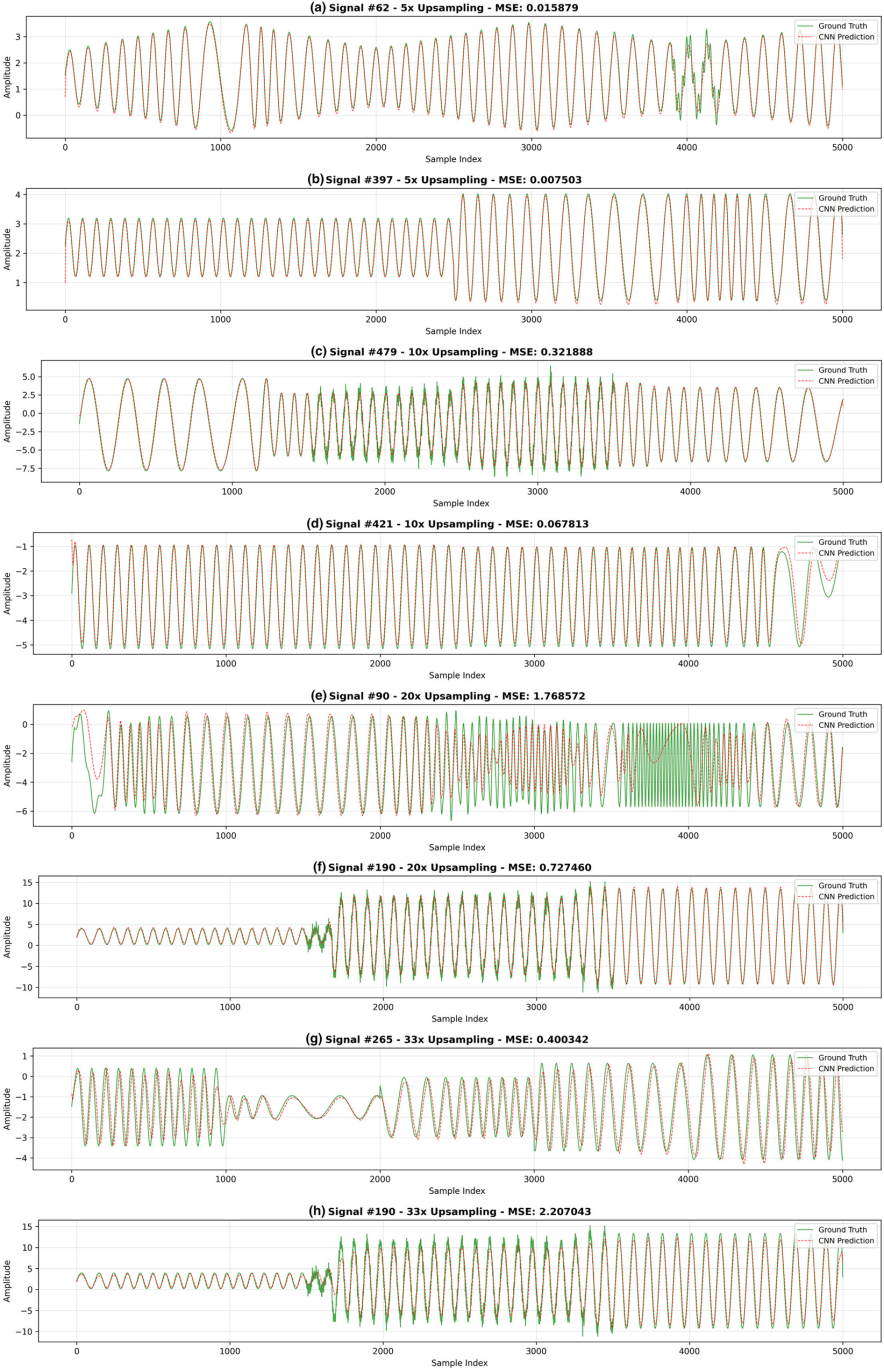
Representative qualitative prediction examples, comparing LR inputs, HR ground truth, and CNN-reconstructed outputs across the multi-scale benchmark. Each panel shows a full-width example.

Representative prediction examples (Fig. 16) provide qualitative comparisons of reconstructed outputs against ground truth across scaling factors. Panels (a–b) correspond to 5× upsampling (1000 → 5000), (c–d) to 10× (500 → 5000), (e–f) to 20× (250 → 5000), and (g–h) to 33× (150 → 5000). Across all factors, the CNN recovers the main waveform structure and overall trend, indicating that the learned mapping is able to exploit consistent LR–HR correlations in the synthetic pairs.

As the upsampling factor increases, the reconstruction problem becomes increasingly ill-posed: fewer observed samples constrain the HR target, and fine temporal details are progressively harder to recover. This is most apparent at 33× , where predictions tend to be smoother and may miss rapid oscillations; correspondingly, a net loss of information at higher frequencies is observed, consistent with the stronger low-pass effect induced by extreme undersampling.

These multi-scale experiments provide quantitative baseline results for future benchmarking studies. In addition to providing a reproducible baseline under a fixed architecture and protocol, the systematic increase in task difficulty—from moderate 5× upsampling to extreme 33× reconstruction—can serve as a practical reference for comparing architectures, loss functions, and training strategies in the time series super-resolution domain. More broadly, CoSiBD enables controlled benchmarking where methods can be compared on identical LR–HR pairs and nuisance settings, supporting fair ablation studies and future community protocols.

### Illustrative Transfer Learning Experiments

To illustrate the application of CoSiBD for training deep learning models, we conducted transfer learning experiments using convolutional neural networks (CNNs) for time-series super-resolution^9,28^. A TimeSeriesSRNet model (encoder-decoder architecture with 1D convolutions: 1 → 64 → 128 → 256, bilinear upsampling, decoder 256 → 128 → 64 → 1) was evaluated on two external signal domains: EEG clinical signals^1^ (500 training, 690 validation samples) and VCTK speech recordings^29^ (44 hours from 109 speakers).

Four training strategies were systematically evaluated: (1) **Reference-only**: trained exclusively on domain-specific data (baseline); (2) **Synth-only**: trained exclusively on CoSiBD synthetic signals; (3) **Mixed**: trained on a combined dataset of synthetic and domain-specific data; and (4) **Tuned**: pre-trained on CoSiBD synthetic data, then fine-tuned on the target-domain training set. Performance was measured using Mean Absolute Error (MAE) between predicted and ground-truth high-resolution signals.

**Fig. 17:**
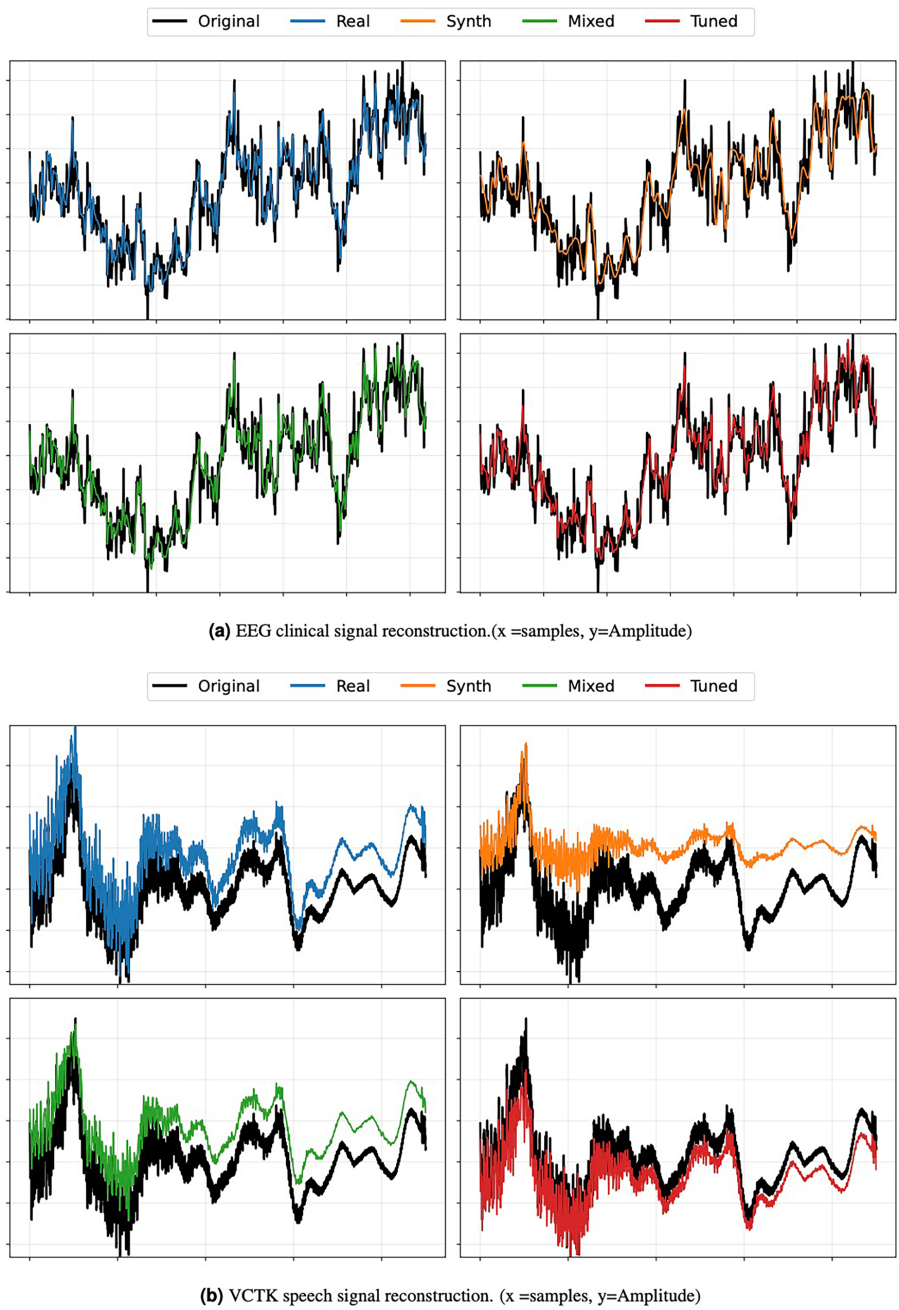
Visual comparison of super-resolution predictions. CoSiBD-enhanced models (Mixed/Tuned) recover finer details in both (**a**) EEG transients and (**b**) speech waveforms compared to the baseline.

The results (Table 6, Fig. 17) show that integrating CoSiBD improves model performance compared to using data of the reference domains alone. While models trained only on synthetic data (Synth-only) yielded higher errors due to domain shift, the **Mixed** and **Tuned** strategies consistently outperformed the Reference-only baseline. In particular, the best EEG result is an MAE of **9.73** achieved by the **Mixed** strategy, while the best VCTK result is an MAE of **4.41** achieved by **Tuned** (Table 6). Specifically, fine-tuning a CoSiBD-pretrained model reduced error significantly on the VCTK dataset.

**Table 6:** Mean Absolute Error (MAE) for CNN-based super-resolution models across training strategies.

Training Strategy	EEG MAE (×10^−2^)	VCTK MAE (×10^−3^)
Reference-only (baseline)	10.77	5.92
Synth-only	12.11	8.79
Mixed (synth + real)	**9.73**	5.59
Tuned (pretrain + finetune)	10.68	**4.41**

Beyond the aggregate MAE values, Fig. 17 provides a qualitative interpretation of these results. In the EEG example, Mixed/Tuned reconstructions better follow fast transients and reduce amplitude under/overshoot compared to the baseline. In the speech example, CoSiBD-enhanced strategies recover sharper oscillatory detail and reduce residual smoothing, visually aligning with the lower MAE reported in Table 6.

All these findings suggest that in two use cases, our dataset contributes to obtaining better results than those obtained using only the reference domains’ data. Anyway, experiments on additional domains are suggested as future research. Detailed experimental methodology and additional comparisons are available in the accompanying repository (see Section of Code availability at the end of the manuscript).

As a further illustration, we applied the TimeSeriesSRNet trained solely on CoSiBD (5× upsampling) to reconstruct full 2-second audio clips from the VCTK corpus without any fine-tuning. The obtained results (mean Pearson correlation *r* = 0.928 between reconstructed and ground-truth signals) suggest that CoSiBD’s frequency and envelope parameters generalize to the signals of the speech dataset considered in the experiments. Reconstructed examples (degraded vs. reconstructed signals under different strategies) are provided in the accompanying repository under AudioModels/results/audio_samples/.

To ensure methodological consistency during validation, both the VCTK audio and EEG datasets were pre-processed following the CoSiBD pipeline. Signals were partitioned into windows of *N* = 5, 000 samples, and their low-resolution counterparts (*M* = 1, 000) were generated using the linear grid re-sampling procedure described in Data Records. In alignment with the benchmark’s design, no anti-aliasing filtration was applied to these external signals, requiring the models to perform reconstruction from raw, aliased observations.

## Usage Notes

The CoSiBD dataset contains high-resolution signals and corresponding subsampled versions at multiple resolutions. Signals are provided in consolidated .txt, .npz, and .json formats. Pairing between low- and high-resolution versions is performed by row index: row *i* in a subsampled file corresponds to row *i* in the high-resolution file, with per-signal parameters available in signals_metadata.json. The dataset is distributed as a single, unified collection without a predefined train/validation/test split. Users should create partitions appropriate to their objectives (e.g., random splits, stratified splits by noise type/level or signal characteristics, cross-validation, or scenario-specific test sets), using the provided metadata to support principled partitioning.

### Reading the Data

In this subsection we show how to read signals stored as consolidated plain text (.txt) files, with one signal per row (samples separated by whitespace). Each file contains multiple time series stacked vertically, where each row corresponds to a single signal. The dataset can be accessed using standard Python tools:


import numpy as np


# Load subsampled (simple decimation) and high-resolution signals # Each .txt file is consolidated: one signal per row x_valid = np.loadtxt(‘SignalBuilderC/data/signals_subsampled_simple_250.txt’) y_valid = np.loadtxt(‘SignalBuilderC/data/signals_high_resolution_5000.txt’)

# Optional: convert to PyTorch tensors # import torch # x_valid = torch.tensor(x_valid, dtype=torch.float32) # y_valid = torch.tensor(y_valid, dtype=torch.float32)

These commands return NumPy arrays (each row corresponds to one signal). Users can optionally convert them to PyTorch tensors.

### Visualizing Signal Pairs

To explore the resolution differences,users can visualize aligned pairs of signals, as indicated in the following example, where well-known functions provided in the Python matplotlib.pyplot interface have been used for that purpose.

import matplotlib.pyplot as plt import numpy as np

# Load subsampled (simple decimation) and high-resolution signals # Each .txt file is consolidated: one signal per row x_lr = np.loadtxt(‘SignalBuilderC/data/signals_subsampled_simple_250.txt’) x_hr = np.loadtxt(‘SignalBuilderC/data/signals_high_resolution_5000.txt’)

# Access a paired signal by row index i = 0 low_res_signal = x_lr[i] high_res_signal = x_hr[i]

# Visualize a paired low- and high-resolution signal plt.figure(figsize=(10, 4))

# High-resolution signal plt.plot(high_res_signal, label='High-resolution (5000 samples)’, alpha=0.8)

# Low-resolution signal (aligned to HR index range for visualization)lr_x = np.linspace(0, len(high_res_signal), len(low_res_signal))plt.scatter(lr_x, low_res_signal, color='red', s=12,label='Low-resolution (250 samples)')

plt.xlabel('Sample index') plt.ylabel('Amplitude') plt.title('Paired Low- and High-Resolution Signal') plt.legend() plt.grid(True) plt.tight_layout() plt.show()

This visualization highlights how the same underlying temporal structure is represented at different resolutions while preserving alignment between paired signals. Additional signal characteristics (e.g., change-points, frequency profiles, or noise configuration) can be retrieved from signals_metadata.json using the same row index.

### Training a baseline model (synthetic-only)

The following example illustrates a minimal synthetic-only training loop for time-series super-resolution using CoSiBD pairs (LR input from simple uniform decimation, HR target). The intent is to provide a compact, reproducible starting point; full training scripts and additional configurations are available in the accompanying repository.

import numpy as np import torch import torch.nn as nn from torch.utils.data import DataLoader, TensorDataset

# Load paired signals (rows align by index) x = np.loadtxt('SignalBuilderC/data/signals_subsampled_simple_250.txt') # (2500, 250) y = np.loadtxt('SignalBuilderC/data/signals_high_resolution_5000.txt') # (2500, 5000)

# Train/val split (example protocol) x_train, y_train = x[:2000], y[:2000] x_val, y_val = x[2000:2500], y[2000:2500]


device = torch.device('mps' if torch.backends.mps.is_available() else 'cpu')


def to_tensor(a): # Convert NumPy array (B, L) into torch tensor (B, 1, L) # The extra channel dimension matches Conv1d input format return torch.tensor(a, dtype=torch.float32).unsqueeze(1) # (B, 1, L)

# Create dataloaders for batched training train_loader = DataLoader(TensorDataset(to_tensor(x_train), to_tensor(y_train)), batch_size=16, shuffle=True) val_loader = DataLoader(TensorDataset(to_tensor(x_val), to_tensor(y_val)), batch_size=16, shuffle=False)

class TinySRNet(nn.Module):def__init__(self, out_len=5000):super().__init__()# Encoder: extract local features in the LR domainself.enc = nn.Sequential(nn.Conv1d(1, 64, kernel_size=5, padding=2), nn.ReLU(),nn.Conv1d(64, 128, kernel_size=5, padding=2), nn.ReLU(),nn.Conv1d(128, 256, kernel_size=5, padding=2), nn.ReLU(),)# Upsampling: map LR features to the HR lengthself.up = nn.Upsample(size=out_len, mode='linear', align_corners=False)# Decoder: project features back to a 1-channel HR signalself.dec = nn.Sequential(nn.Conv1d(256, 128, kernel_size=5, padding=2), nn.ReLU(),nn.Conv1d(128, 64, kernel_size=5, padding=2), nn.ReLU(),nn.Conv1d(64, 1, kernel_size=5, padding=2),)

def forward(self, x): z = self.enc(x) z = self.up(z) return self.dec(z)

model = TinySRNet(out_len=5000).to(device) # Optimizer and loss (MSE is a standard regression objective) opt = torch.optim.Adam(model.parameters(), lr=1e-3, weight_decay=1e-5) loss_fn = nn.MSELoss()

for epoch in range(1, 11): model.train() for xb, yb in train_loader: xb, yb = xb.to(device), yb.to(device) # Forward + loss + backward + update opt.zero_grad() pred = model(xb) loss = loss_fn(pred, yb) loss.backward() opt.step()

model.eval() with torch.no_grad(): # Validation loop: average loss over batches val_loss = 0.0 for xb, yb in val_loader: xb, yb = xb.to(device), yb.to(device) val_loss += loss_fn(model(xb), yb).item() val_loss /= len(val_loader) print(f”epoch=epoch:02d val_mse=val_loss:.4f”)

## Data Availability

The dataset itself is published separately at the Zenodo repository^20^ and all related data files are publicly accessible at the following link: https://zenodo.org/records/18295713 (10.5281/zenodo.18295713). The Zenodo record distributes the dataset under the Creative Commons Attribution 4.0 International (CC BY 4.0) license.

## Code availability

The complete signal generation pipeline, including modules for frequency profile generation, amplitude envelope construction, spline interpolation, noise application, and data export in multiple formats, is available at: https://github.com/DhamarAM/SignalBuilder/tree/main.

The repository includes SignalBuilderC, a modular Python package with documented functions for: (1) generating high-resolution signals with configurable parameters, (2) creating subsampled versions via simple decimation (uniform subsampling), (3) exporting signals in NumPy, text, and JSON formats, and (4) comprehensive metadata generation. All code is provided with example notebooks demonstrating dataset regeneration and usage. These scripts are distributed under the MIT License.
